# Parent‐Adolescent Digital Communication and Relationship Quality in the United States: A Systematic Review

**DOI:** 10.1002/jad.70233

**Published:** 2026-07-20

**Authors:** Eunyoung Park, Jodi Dworkin

**Affiliations:** ^1^ Department of Family Social Science University of Minnesota St Paul Minnesota USA

**Keywords:** adolescents, digital communication, parents, PRISMA, relationship quality

## Abstract

**Introduction:**

Despite the growing centrality of digital communication in family life, little is known about its role in parent‐adolescent relationships, especially when parents and adolescents share a household. This review was designed to synthesize U.S.‐based research on digital communication and relationship quality in these families across theoretical foundations, conceptualizations, measurements, and associations.

**Methods:**

We conducted systematic searches following the PRISMA guidelines in four online databases: Academic Search Premier, Google Scholar, PsycINFO, and Web of Science. Based on the predefined eligibility criteria, the search yielded 10 studies. The methodological quality of included studies was assessed.

**Results:**

Across studies spanning multiple disciplines, digital communication was most often conceptualized and measured as frequency of use across different modalities (text messaging, voice calls, social media), while parent‐adolescent relationship quality was captured along cognitive and emotional dimensions. Findings on the association between digital communication and relationship quality were inconsistent. This inconsistency appeared to stem in part from how the constructs were defined and measured, and from the use of frequency‐based approaches that aggregated communication serving different purposes.

**Conclusion:**

This review demonstrates that digital communication plays a distinct supplementary role in parent‐adolescent relationships, bridging physical and emotional distance rather than replacing face‐to‐face interaction. Its influence is shaped less by how often communication occurs and more by its purpose, the modalities used, adolescents' device ownership, and the gender composition of the dyad. By synthesizing how parent‐adolescent digital communication has been theorized, conceptualized, and measured, this review provides a foundation for future research on digitally mediated family life.

Smartphones offer a wide range of digital communication features, and adolescents actively use them for entertainment and to connect with friends and family (Allaby and Shannon 2020; Radesky et al. 2023); 95% of U.S. adolescents aged 13 to 17 have access to a smartphone (Faverio and Sidoti 2024). In fact, adolescents report using smartphones as their constant companions in their daily lives (Radesky et al. 2023). This active engagement has significantly changed the way parents and adolescents communicate, influencing adolescent well‐being (e.g., Coyne et al. 2020) and family dynamics (e.g., Jensen et al. 2021b; Tammisalo and Rotkirch 2022). Unfortunately, a comprehensive understanding of how adolescents and their parents integrate digital communication into their relationships is challenged by rapid changes in the use of digital devices and applications and the lack of a unified conceptualization across studies. This systematic review was designed to synthesize the association between parent‐adolescent digital communication and relationship quality by analyzing the theoretical frameworks and measures used in existing studies. We specifically focused on U.S. adolescents living with their parents, recognizing that adolescents in alternative living arrangements, such as foster care or independent living, experience fundamentally different family dynamics compared to those living with their parents. This targeted approach supports a more contextualized understanding of how adolescents and their parents communicate through digital devices, and how the nature and impact of such communication may vary across relationships. We view this review as just one step toward providing research‐based guidance to families, practitioners, and researchers on how digital communication can be leveraged to support healthy family relationships.

Digital communication encompasses communication using a variety of digital modalities, such as texting, calling, video calling, and social media interactions, as well as various devices, including smartphones, computers, and tablets. Adolescents are avid users of digital communication; 96% of U.S. adolescents report daily internet use, and approximately 46% report being online “almost constantly,” up from 24% a decade ago (Faverio and Sidoti 2024). Specifically, adolescents use digital communication, such as social media and texting, to cultivate interpersonal relationships, including maintaining connections that supplement in‐person interactions (Anderson et al. 2022; Ehrenreich et al. 2021). Further, digital communication also significantly shapes how adolescents interact with their parents. Current research on parent‐adolescent digital communication primarily focuses on the frequency or quantity of calls (Jensen et al. 2021a; Manago et al. 2020), texts (Janssen et al. 2025; Jensen et al. 2021a, 2021b; Manago et al. 2020), and social media use (Janssen et al. 2025) for different topics, including support seeking, coordination, and communication about logistics.

Given the reciprocal nature of communication between adolescents and their parents (Branje et al. 2012; Sherman et al. 2013), digital communication may either strengthen or weaken the parent‐adolescent relationship. Ribak (2009) conceptualizes the mobile phone as a developmental “transitional object” that functions both as a remote control through which parents extend supervision and as a digital umbilical cord that helps sustain connection and security as adolescents negotiate growing autonomy. This framing suggests that the same channel can be experienced as both surveillance and support, helping explain why digital communication may both strengthen and weaken parent‐adolescent ties.

Relationship quality between parents and adolescents is typically conceptualized along two dimensions: positive features such as warmth, support, closeness, and open communication, and negative features such as conflict, control, and perceived intrusiveness (Boele et al. 2019; Laursen and Collins 2009). Because adolescence is marked by the simultaneous pursuit of autonomy and continued dependence on parents (Steinberg 2001; Steinberg and Silk 2002), everyday communication plays a key role in shaping the parent‐adolescent relationship.

Given the supportive role of technology in parent‐adolescent relationships, digital communication enables adolescents to easily stay connected with family members (Fletcher et al. 2018) in ways that are independent of time or location (Manago et al. 2020). Specifically, adolescents' digital communication allows for increased and immediate communication regardless of physical distance from parents (Rudi et al. 2014; Warren and Aloia 2018). Maintaining constant connectivity through digital communication can lead to an increased perception of time spent together, which consequently strengthens the emotional bond (Devitt and Roker 2009). Further, being able to choose and utilize different types of digital interactions (e.g., texting and calling) can strengthen bonds with parents (Moawad and Ebrahem 2016). Despite its role in supplementing in‐person interactions, frequent digital communication between parents and adolescents can give rise to relationship challenges, including concerns around privacy and autonomy. Existing research highlights considerable discrepancies in how parents and adolescents perceive digital communication, with behaviors parents consider supportive often experienced by adolescents as intrusive or overreaching (Chang 2015; Racz et al. 2015). Adolescents' own perceptions of parental digital involvement are therefore central to understanding these dynamics, as the line between involvement and overinvolvement is shaped not only by individual relationship factors but also by broader family values and norms around autonomy, privacy, and parental authority.

Beyond these relational and cultural factors, parents also differ in their capacity to engage with digital tools. Parents' familiarity with and experience of digital communication vary substantially across families, shaped by factors such as generations, socioeconomic background, and individual experiences (Navarro and Tudge 2023; Rogers et al. 2021; Zhao et al. 2023). As a result, some parents may struggle to use digital tools effectively with their adolescents, find it challenging to adapt these tools to family communication, or have difficulty navigating the nuances of digital devices for communication. This variability may create challenges regarding what to communicate through digital devices and, if using digital devices, which modalities to use, depending on what is being communicated (Barrie et al. 2019). Understanding these communication dynamics is essential for developing context‐sensitive strategies that help families effectively integrate digital tools into family life while maintaining healthy parent‐adolescent relationships.

In the face of mixed empirical evidence from existing research, there is a compelling need for a review specifically focused on how adolescents use different digital communications with their parents and how these interactions are associated with their relationship quality. This systematic review addresses this knowledge gap by concentrating on the daily communication of U.S. adolescents and their parents in shared living arrangements. We conducted a systematic review to critically analyze and synthesize foundational theories, conceptual frameworks, conceptualizations, measures, and their associations. Given that scholars across diverse disciplines, including family science, psychology, media, and communication, have investigated this topic, an interdisciplinary approach was used to consolidate the empirical evidence. In addition, cultural context shapes digital communication in important ways, including differences in digital access, skills, and family rules around technology use (Calderon Gomez 2021; Robinson et al. 2015; Tadpatrikar et al. 2021). Because these factors limit generalizability across cultural settings, we are explicit throughout about our inclusion criteria and the limits on generalizing beyond this population. This comprehensive review can support social scientists and researchers across disciplines in identifying and developing frameworks and models to support future interdisciplinary research aimed at strengthening family relationships in the digital age.

## 1 Methods

### 1.1 Literature Search Strategy

This systematic review adhered to the updated reporting guidelines based on the Preferred Reporting Items for Systematic reviews and Meta‐Analyses (PRISMA; Page et al. 2021). An initial systematic search was conducted in March 2023 and updated through June 2026. The search was conducted using four databases: Academic Search Premier, Google Scholar, PsycINFO, and Web of Science. The fundamental search terms (See Table 1) included four criteria: sampling (e.g., adolescents), relationship (e.g., parents), digital communication (e.g., computer‐mediated communication, smartphone), and relationship quality (e.g., closeness, connectedness). The search was conducted for studies published within the last 13 years (2011–2024), given that three‐quarters (77%) of adolescents aged 12 to 17 had a cell phone in 2011, a notable increase from late 2004, when only 45% of adolescents had a cell phone (Lenhart 2012). The age range of 12 to 17 was selected as the primary focus, as this period aligns with established definitions of adolescence (Curtis 2015) and coincides with widespread cell phone adoption among this age group (Lenhart 2012), making it a particularly relevant window for examining technology's role in parent‐adolescent relationships. We distinguish this developmental stage from emerging adulthood (ages 18–29), which is characterized by its own distinct relational and developmental dynamics (Arnett 2000). The search encompassed study titles, abstracts, and keywords. All retrieved literature was screened for duplicates, which were identified and removed. Lastly, title and abstract screenings were performed to assess eligibility criteria (Figure 1).

**Table 1:** Search terms.

Criteria	Search terms
Sampling	“early adolescence,” “adolescen*,” “youth,” “teenager*”
Relationship	“parent*,” “mother,” “father,” “family,” “parent‐child relation*,” “maternal,” “paternal”
Digital communication	“computer‐mediated communication,” “electronic communication,” “Information and Communication Technology,” “mobile phone,” “cell phone,” “smartphone,” “texting,” “social media,” “digital communication”
Relationship quality	“relationship quality,” “interpersonal interaction,” “interpersonal relationship,” “relationship satisfaction,” “closeness,” “connectedness,”

**Figure 1:**
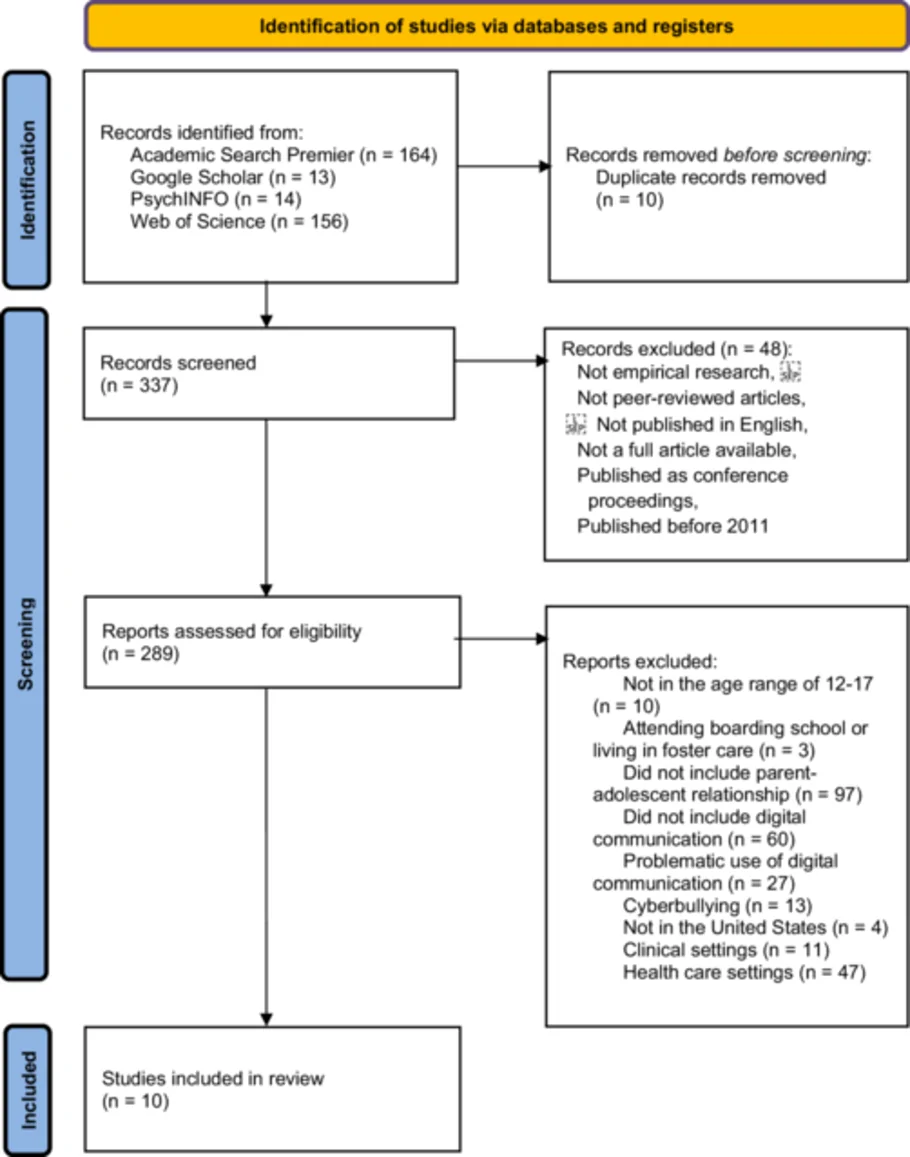
PRISMA flow diagram showing the process of article selection for inclusion in the systematic review.

### 1.2 Eligibility Criteria

Studies were included if they satisfied the following criteria: (1) Studies were empirical and published in English in peer‐reviewed scholarly journals; (2) Studies focused on the experience of adolescents between the ages of 12 and 17 and their parents; (3) Studies focused on daily communications between parents and adolescents living together; (4) Studies assessed the quality of parent and adolescent relationships such as closeness, communication, and spending time together; (5) Studies pertained to communication experiences using digital devices; (6) Studies collected data from the United States, to avoid confounding findings with cross‐cultural differences in digital device ownership, usage, and cultural variations in digital communication within parent‐adolescent relationships.

Exclusion criteria were as follows: (1) Studies were not published in English (i.e., Spanish), not peer‐reviewed (i.e., dissertations, book chapters), and were not reporting on empirical research; (2) Study participants were not in the age range of 12–17; (3) Studies examined only specific family circumstances, such as migrant families, youth attending boarding school or living in foster care; (3) Studies assessed relationships with friends, between couples, or other family members, excluding parent‐adolescent relationships; (4) Studies did not include digital communication or focused only on problematic digital communication or cyberbullying; (5) Studies were conducted outside the United States; (6) Studies focused on clinical or healthcare settings.

### 1.3 Quality Assessment

The quality of eligible studies was assessed using the Mixed Methods Appraisal Tool (MMAT; Hong et al. 2018). The MMAT was selected for this review because of its unique capacity to identify specific methodological strengths and limitations across the included studies using a single, unified framework. The MMAT begins with two screening questions intended to establish the adequacy of each study: “Are there clear research questions?” and “Do the collected data allow addressing the research questions?” If the response to at least one of these questions is “No” or “Can't Tell,” further appraisal is deemed unfeasible or inappropriate, as a study lacking conceptual or methodological clarity cannot be meaningfully evaluated against substantive quality criteria. In the current review, all studies were determined to have sufficient conceptual and methodological clarity for evaluation.

Studies that passed the initial screening were then assessed using design‐specific criteria tailored to their respective methodologies (Table 2): quantitative studies (*n* = 9) and mixed‐method (*n* = 1). These criteria address the core elements of methodological rigor relevant to each design. Each criterion is rated as Yes, No, or Can't Tell, with scores of 1, 0, and 0, respectively, and summed to produce an overall quality score. Studies were subsequently classified as high (100% Yes), moderate (60%–80% Yes), or low quality (below 60% Yes). This scoring approach provides a transparent basis for quality judgments.

**Table 2:** Quality assessment table for quantitative and mixed methods studies.

Quantitative study (Author, Year)	Quantitative descriptive					Score
	1	2	3	4	5	
Aloia and Warren (2019)	Yes	Yes	Yes	Yes	Yes	High
Jensen et al. (2021a)	Yes	Yes	Yes	Yes	Yes	High
Jensen et al. (2021b)	Yes	Yes	Yes	Yes	Yes	High
Manago et al. (2020)	Yes	Yes	Yes	Yes	Yes	High
Padilla‐Walker et al. (2012)	Yes	Yes	Yes	Yes	Yes	High
Vaterlaus et al. (2019)	Yes	Yes	Yes	Yes	Yes	High
Warren and Aloia (2018)	Yes	Yes	Yes	CT	Yes	Moderate
Weisskirch (2011)	Yes	Yes	Yes	No	Yes	Moderate
Williams and Merten (2011)	Yes	Yes	Yes	No	Yes	Moderate
Mixed methods	Quantitative descriptive					Score
Study (Author, Year)	1	2	3	4	5	
Fletcher et al. (2018)	Yes	Yes	Yes	Yes	Yes	High
	Qualitative					
	1	2	3	4	5	
	Yes	Yes	Yes	Yes	Yes	
	Mixed methods					
	1	2	3	4	5	
	Yes	Yes	Yes	Yes	Yes	

*Note:* Response options: Yes for 1; No or Can't tell (CT) for 0. Quantitative Descriptive questions: (1) Is the sampling strategy relevant to address the research question? (2) Is the sample representative of the target population? (3) Are the measurements appropriate? (4) Is the risk of nonresponse bias low? (5) Is the statistical analysis appropriate to answer the research question? Qualitative questions: (1) Is the qualitative approach appropriate to answer the research question? (2) Are the qualitative data collection methods adequate to address the research question? (3) Are the findings adequately derived from the data? (4) Is the interpretation of results sufficiently substantiated by data? (5) Is there coherence between qualitative data sources, collection, analysis, and interpretation? Mixed Methods questions: (1) Is there an adequate rationale for using a mixed methods design to address the research question? (2) Are the different components of the study effectively integrated to answer the research question? (3) Are the outputs of the integration of qualitative and quantitative components adequately interpreted? (4) Are divergences and inconsistencies between quantitative and qualitative results adequately addressed? (5) Do the different components of the study adhere to the quality criteria of each tradition of the methods involved? Scores are rated as high (100%), moderate (60‐80%), or low (below 60%).

## 2 Results

### 2.1 Study Selection

The initial searches identified a total of 347 studies. After removing duplicates (*n* = 10), the titles and abstracts of 337 studies were screened for relevance, and 48 records were excluded for not meeting the inclusion criteria of peer‐reviewed empirical studies published within the specified time period. Following this, 289 studies were selected for full‐text review; based on the scope of this review, 279 were excluded when assessed against the eligibility criteria. This led to the identification and inclusion of 10 studies. The entire selection process is presented in the PRISMA flowchart (Figure 1).

### 2.2 Quality Assessment

The quality assessment, using MMAT ratings for each study, is presented in Table 2. Using the system of summed scores for classification, all studies were eligible for further appraisal, and the results of the MMAT deemed six studies as high in quality (Aloia and Warren 2019; Jensen et al. 2021a, 2021b; Manago et al. 2020; Padilla‐Walker et al. 2012; Vaterlaus et al. 2019) and three studies as moderate in quality (Warren and Aloia 2018; Weisskirch 2011; Williams and Merten 2011). Studies of moderate quality lacked explanations of sampling strategies (Warren and Aloia 2018) and reported high rates of nonparticipation (e.g., over 50%), which can increase the risk of nonresponse bias (Weisskirch 2011; Williams and Merten 2011).

### 2.3 Study Characteristics

The characteristics of the included studies are tabulated in Table 3. The publication period ranged from 2011 to 2024, with 70% of the studies being published from 2018 to 2021. Notably, no studies meeting our inclusion criteria were published between 2022 and 2024. The eligible studies included quantitative (*n* = 9) and mixed‐method (*n* = 1) designs. Six quantitative studies used a cross‐sectional design, and three used an intensive longitudinal design. One study employed a mixed‐methods design, integrating findings from interviews with quantitative data.

**Table 3:** Characteristics of Included Studies.

	Age					Digital communication		Relationship Quality	
Study (Author, Year)	Adolescent	Parent	Sample size	Study design	Analytic approach	Measure	Modalities	Measure	Dimensions
Aloia and Warren (2019)	12–17 (*M* = 14.54)	25‐74 (*M* = 43.79)	504 dyads	C	Univariate Correlation	Frequency and reasons for digital communication (Kennedy et al. 2008)	Mobile phone	Quality of Relationships Inventory (Pierce 1994)	Cognitive
Fletcher et al. (2018)	14–18 (*M* = 16)		41 adolescents	M	Descriptive, Coding	Cell phone histories	Text, call	Semi‐structured Interview	
Jensen et al. (2021a)	10–17 (*M* = 13.37)		388 adolescents	L	Multilevel modeling	Daily frequency of digital communication, developed for this study	Text or call	Parent hassles and uplifts (Ham and Larson 1990; Kearney et al. 1993; Shahar et al. 2003; Evans et al. 2009)	Cognitive, emotional
Jensen et al. (2021b)	10–17 (*M* = 13.37)		388 adolescents	L	Multilevel modeling	Daily frequency of text and estimated hours for digital communication	Text, call, online	Parent hassles and uplifts Ham and Larson 1990; Kearney et al. 1993; Shahar et al. 2003; Evans et al. 2009), time spent communicating with parents	Cognitive, emotional
Manago et al. (2020)	14–18 (*M* = 16.70)		169 adolescents	L	Regression	Daily and weekday hours spent on digital communication, Daily frequency of texts, developed for this study	Text, call, video call	Emotional Reliance Scale (Ryan et al. 2005)	Emotional
Padilla‐Walker et al. (2012)	13–16 (*M* = 14.32)		453 adolescents	C	Structural equation modeling	Frequency of family media use, developed for this study	Text or call, e‐mail, or social networking sites	Parenting Styles and Dimensions Questionnaire‐Short Version (Robinson et al. 2001)	Cognitive, emotional
Vaterlaus et al. (2019)	16–25 *(M*_*Adolescent*_ = 17.19, *M*_*Young Adult*_ = 19.80)	*M*_Mother_ = 47.57, *M*_Father_ = 49.56	766 adolescents and young adults	C	Regression	Quality of interactions using digital communication, developed for this study	Text, call, e‐mail, social networking, video call	Parent‐adolescent Relationship Quality (Fallon and Bowles 1997)	Cognitive, emotional
Warren and Aloia (2018)	12–17 (*M* = 14.54)	25‐74 (*M* = 43.73)	504 dyads	C	Multiple mediation analyses	Frequency and reasons for digital communication (Kennedy et al. 2008)	Mobile phone, email, text, instant message, social media	Unidimensional Relationship Closeness Scale (Dibble et al. 2012)	Cognitive
Weisskirch (2011)	14–19 (*M* = 16.25)	25‐66 (*M* = 45.38)	196 dyads	C	Univariate Correlation	Frequency of initiated calls, developed for this study	Call	Adolescent Family Process (Vazsonyi et al. 2003)	Cognitive
Williams and Merten (2011)	12–17	*M* = 41.21	386 parents	C	Regression	Internet use at home and Internet co‐usage (Kennedy et al. 2008)	Internet	Perceived family connectedness, developed for this study	Cognitive, emotional

*Note:* C, Cross‐sectional; L, Longitudinal; M, Mixed‐method.

The sample across all studies consists of adolescents aged 10 to 25 years and their parents, aged 25 to 74 years. Although the primary focus of this review is on adolescents aged 12 to 17, four studies (Fletcher et al. 2018; Manago et al. 2020; Vaterlaus et al. 2019; Weisskirch 2011) included older participants whose ages extended into emerging adulthood (18 to 25). Specifically, Vaterlaus et al. (2019) included both adolescents and emerging adults in their original sample; only the adolescent subsample was retained for this review. This age distinction is important, as adolescence and emerging adulthood are each marked by distinct shifts in autonomy (Steinberg and Silk 2002), identity formation (Erikson 1968), and the renegotiation of parent‐child relationships (Steinberg 2001). As adolescents move toward greater independence, parental authority is increasingly renegotiated (Steinberg 2001), and these shifts may significantly influence how young people respond to and engage in digital dialogue with their parents.

Beyond age, gender is another dimension along which the reviewed studies varied. Of the 10 studies, only four (Aloia and Warren 2019; Fletcher et al. 2018; Vaterlaus et al. 2019; Weisskirch 2011) examined gender differences within parent‐adolescent dyads. Given that gender differences in parent‐adolescent dyads influence communication styles and emotional dynamics (Branje 2018; Yang et al. 2025), gender may influence how digital communication is experienced and interpreted through parent‐adolescent interactions.

### 2.4 Foundational Theories and Conceptual Frameworks

#### 2.4.1 Family Theories

Two theoretical frameworks were recurring across this body of work, and both treat digital communication as a force acting on the family rather than as a neutral channel. The first, family systems theory, anchored three studies (Aloia and Warren 2019; Padilla‐Walker et al. 2012; Warren and Aloia 2018). These studies conceptualize digital communication as an external influence capable of either strengthening or straining parent‐adolescent interaction. Padilla‐Walker et al. (2012) emphasized the positive potential in shared experience, highlighting that media operates as an “outside source of influence on the family (p. 428),” that can support repeated interactions, such as family rituals or celebrations, and through them, connection. Warren and Aloia (2018) attempted to explain the specific reasons behind parent‐adolescent digital communication, while Aloia and Warren (2019) highlighted the openness and continuity that digital communication affords in family interaction. These studies use family systems theory to frame digital communication as a source of shared experiences that provides regulation and accessibility to family communication and closeness.

The second framework, Bronfenbrenner's (1977, 1979, 1994) ecological systems theory, informed two studies (Vaterlaus et al. 2019; Williams and Merten 2011) that consider digital communication within the family microsystem itself rather than outside it. Both studies conceptualize digital communication as a means of facilitating bidirectional exchange among family subsystems, which can strengthen bonds (Vaterlaus et al. 2019; Williams and Merten 2011). Both also extend the analysis outward, treating broader societal changes as a macro‐level pressure that shapes how digital communication enters and operates within the family as a microsystem. These two frameworks are perhaps complementary. Ecological systems theory asks how digital communication alters interaction within the family, while family systems theory allows consideration of how forces beyond the family shape that communication in the first place.

#### 2.4.2 Parenting Frameworks

Two studies (Jensen et al. 2021a; Weisskirch 2011) employed approaches that emphasize how parents use digital communication as a tool for parenting, though they conceptualize parenting itself in different terms. Jensen et al. (2021a) adopted a co‐constructionist approach (Subrahmanyam et al. 2006), which holds that adolescents' online and offline worlds are interconnected, such that interpersonal processes are expected to operate similarly across digital and in‐person contexts rather than operating in unique ways across these different domains. Consistent with this premise, Jensen et al. (2021a) emphasized that parenting behaviors were equally effective whether enacted in person or through digital communication. Weisskirch (2011), by contrast, employed a dimensional approach (Steinberg and Silk 2002), which conceptualizes parenting as a set of distinct dimensions that can each independently influence adolescent development. Reflecting this framework, Weisskirch (2011) identified three components of parenting (i.e., harmony, autonomy, and conflict) and examined how each operated within digital interactions. Together, these studies illustrate two complementary ways of locating parenting in digital spaces, both as a context for parenting in real life and as a medium through which specific parenting dimensions are enacted.

#### 2.4.3 Media and Communication Theories

Three studies (Fletcher et al. 2018; Jensen et al. 2021b; Manago et al. 2020) drew on media and communication theories. However, they each centered on a different modality and motivation for digital communication in parent‐adolescent relationships. Fletcher et al. (2018) approached digital communication through uses and gratification theory (Ruggerio 2000), focusing on how adolescents use calls and texts to satisfy their needs for contact with their parents. In contrast, guided by the displacement hypothesis (Kraut et al. 1998), Jensen et al. (2021b) framed digital communication as a potential cost to the relationship rather than a benefit to ask whether time spent communicating digitally displaces in‐person opportunities for connection. Manago et al. (2020) integrated several frameworks at once, using and gratifications theory (Katz et al. 1973), media multiplexity theory (Haythornthwaite 2005), and the hyperpersonal model (Walther 1996), to suggest that using multiple digital modalities can deepen an already strong parent‐adolescent bond by motivating parents and adolescents to stay in contact and optimizing opportunities for disclosure and intimacy. Taken together, these studies show media theories being used less to settle the debate of whether digital communication helps or harms the parent‐adolescent relationship and more to specify the conditions and mechanisms, such as need, displacement, modality, and existing closeness, under which it might do both.

### 2.5 Measures

#### 2.5.1 Digital Communication

Consistent with the definition outlined in the literature review, each included study operationalized digital communication according to its own conceptualization, and these variations are summarized in Table 4. The modalities of digital communication included text and/or call (*n* = 3), text, call, and video call (*n* = 1), call (*n* = 1), multiple digital communication modalities (e.g., email, social networking sites, instant message; *n* = 3), Internet (*n* = 1), and general mobile phone use (*n* = 1). Furthermore, digital communication was evaluated based on both the frequency of daily use and the reasons for its use. Specifically, five studies (Fletcher et al. 2018; Jensen et al. 2021b; Manago et al. 2020; Padilla‐Walker et al. 2012; Williams and Merten 2011) examined the frequency of digital communication using a self‐reported Likert scale (i.e., a 7‐point scale ranging from *Never* to *Several times a day*) and self‐reported an exact number of texts and hours of calls throughout the day. Four studies (Aloia and Warren 2019; Jensen et al. 2021a; Warren and Aloia 2018; Weisskirch 2011) assessed both the frequency and the reasons (i.e., coordination, support, conflict, sharing) for adolescents' use of specific modalities. One study (Vaterlaus et al. 2019) used a measure of the quality of interaction experienced through each modality of digital communication.

**Table 4:** Conceptualization of digital communication and relationship quality.

Study (Author, Year)	Conceptualization of digital communication	Conceptualization of relationship quality
Aloia and Warren (2019)	Frequency and reasons (planning behaviors, comforting messages, material sharing) of using a mobile phone	Parental support, relational depth, parental conflict
Fletcher et al. (2018)	Frequency of adolescents' calls and texts to interact with their parents	Emotional connection, managerial parenting, perceptions of closeness
Jensen et al. (2021a)	Frequency of adolescents' calls and texts to support seeking, solicitation, disclosure, and control	Parents' uplifts and hassles
Jensen et al. (2021b)	Number of texts sent and duration (hours) of digital communication use	Parent hassles and uplifts, time spent communicating with parents
Manago et al. (2020)	Frequency of texts and time spent on calls and video calls	Emotional support
Padilla‐Walker et al. (2012)	Frequency of using cell phones, email, and social networking sites with family members	Parental warmth and support
Vaterlaus et al. (2019)	Quality of parent‐child interaction experienced through texts, calls, e‐mail, social networking, and video calls	Emotionally being together to talk about things that are important to the child, feeling safe to ask questions, and feeling comfortable when discussing things that the child would not want any other person to know
Warren and Aloia (2018)	Frequency and reasons (coordination, support, conflict, sharing) of using mobile phone, email, text, instant message, social media	Perceived closeness between adolescents and their parents affects and is affected by each other
Weisskirch (2011)	Frequency and reasons (ask, confer, and social support seeking) of initiated calls	Dimensions of parenting (closeness, monitoring, support, communication, conflict, and peer approval)
Williams and Merten (2011)	Frequency of Internet use at home and co‐usage with parents	Perceived family connectedness (time spent, closeness, connectedness, communication quality)

However, it is important to assess both the frequency of digital communication and the underlying reasons for using it. Frequency often varies depending on the reasons, which reflect differing needs, intentions, and relational dynamics. Seven studies (Aloia and Warren 2019; Jensen et al. 2021a; Padilla‐Walker et al. 2012; Vaterlaus et al. 2019; Warren and Aloia 2018; Weisskirch 2011; Williams and Merten 2011) used self‐report measures to assess the daily frequency of adolescents' digital communication with their parents. Among them, two studies (Aloia and Warren 2019; Warren and Aloia 2018) employed established and validated measures of mobile communication frequency (Kennedy et al. 2008). Three studies (Fletcher et al. 2018; Jensen et al. 2021b; Manago et al. 2020) used objective measurements to examine daily digital communication, including the number of texts and hours adolescents spent calling and posting on social media. This highlights the different approaches to measuring digital communication, from self‐reports to objective assessments, and the application of validated measures.

Only one study (Weisskirch 2011) required both parent and adolescent participants to have their own device for digital communication (i.e., cellular phone) for at least 6 months prior to participating in the study. Instead, four studies (Aloia and Warren 2019; Jensen et al. 2021a, 2021b; Manago et al. 2020) examined only whether adolescents owned their own devices (i.e., cell phones, mobile phones, personal smartphones with Internet access). Specifically, each study provided the percentage of adolescents who owned or used their own device, ranging from 49.9% to 91.5%. Furthermore, two studies (Jensen et al. 2021a, 2021b) described a device that could only be used to collect data (i.e., not equipped with capabilities for texting and calling). Due to the nature of digital communication, adolescents' ownership might influence how they communicate and maintain relationships with their parents. For example, ownership may impact knowing how to use multiple functions, such as sending and receiving emojis, tapping back, and posting and liking posts on social media. Thus, it is important to describe their ownership and the duration of their use of digital communication.

#### 2.5.2 Relationship Quality

Consistent with the relationship quality conceptualizations presented in Table 4, nine studies used self‐report measures, while one study employed semi‐structured interviews to assess different aspects of parent‐adolescent relationship quality. Three studies (Aloia and Warren 2019; Warren and Aloia 2018; Weisskirch 2011) focused on the cognitive aspects, such as soliciting advice and discussing roles and responsibilities. One study (Manago et al. 2020) placed greater emphasis on the emotional aspect, including attachment and perceived emotional closeness. Five studies (Jensen et al. 2021a, 2021b; Padilla‐Walker et al. 2012; Vaterlaus et al. 2019; Williams and Merten 2011) aimed to capture both cognitive and emotional dimensions, including parental warmth and support. Lastly, one study (Fletcher et al. 2018) employed open‐ended questions to take a broad look at relationship quality: “When communicating with your parents using your cell phone, do you tend to call them more or text them more? Why do you think this is?” and “Can you give me some examples of when you used your cell phone to communicate about…?” Digital communication plays a unique role in shaping the quality of these relationships through various characteristics and effects. Given that the parent‐adolescent relationship is bidirectional and multidimensional, it is crucial to specify which aspects of relationship quality are examined and to account for the specific modalities of digital communication used in order to accurately capture its role in the relationship.

### 2.6 The Role of Digital Communication in Shaping Parent‐Adolescent Relationship Quality

Seven studies investigated the association between the frequency of digital communication and parent‐adolescent relationship quality, yielding mixed findings. While six studies found that adolescents who reported more frequent digital communication tended to report better relationship quality with their parents (Fletcher et al. 2018; Jensen et al. 2021a; Manago et al. 2020; Padilla‐Walker et al. 2012; Warren and Aloia 2018; Weisskirch 2011), one found no associations (Jensen et al. 2021b). Similar results were found when considering parents' perspectives. Four studies found that more frequent parental digital communication was associated with better relationship quality as perceived by adolescents (Aloia and Warren 2019; Padilla‐Walker et al. 2012; Warren and Aloia 2018; Williams and Merten 2011). This picture is complicated, however, by evidence that the purpose of communication matters as much as its frequency: one study found that less frequent parental calls and texts to handle conflict were associated with better relationship quality (Warren and Aloia 2018). Taken together, these mixed results make it difficult to draw simple conclusions about how the overall use of digital communication between parents and adolescents relates to their relationship quality.

Beyond overall frequency, the specific modality of digital communication appears to matter. Three studies have found that more frequent texting was associated with adolescents' perceptions of greater closeness with parents (Manago et al. 2020; Vaterlaus et al. 2019) and a stronger family connection (Padilla‐Walker et al. 2012). Vaterlaus et al. (2019) found that adolescents who texted their parents more often reported feeling more connected and spending more time with both mothers and fathers. Yet the same study found a contrasting pattern for social networking sites: more frequent use with parents was associated with lower relationship quality with fathers. Vaterlaus et al. (2019) posited that brief, passive updates on social media may create a sense of being “caught up,” potentially reducing more substantive father‐child interaction through other channels. Four studies (Aloia and Warren 2019; Jensen et al. 2021a; Warren and Aloia 2018; Weisskirch 2011) examined the reasons behind digital communication and how they relate to parent‐adolescent relationship quality. A consistent theme emerged: the purpose of contact shapes whether it helps or harms the relationship. Communication aimed at expressing support was associated with better relationship quality with parents (Warren and Aloia 2018). In contrast, communication used for planning and sharing materials (Aloia and Warren 2019), handling conflicts (Warren and Aloia 2018), and seeking support or managing parental control (Jensen et al. 2021a) was associated with lower relationship quality, while content sharing and coordination showed no associations (Warren and Aloia 2018). A similar logic appears in Weisskirch (2011), where the purpose of communication, whether for conflict or closeness, was differentiated. Specifically, parental calls for monitoring and tracking schoolwork, or calls made when parents were upset, were positively associated with parent‐adolescent conflict (Weisskirch 2011). Taken together, these findings reinforce the broader pattern that both the modality and the underlying purpose of digital communication, rather than its frequency alone, shape how adolescents perceive their relationship with their parents.

## 3 Discussion

As digital communication has become an essential part of adolescents' lives and added a new dynamic to family relationships, researchers have focused on how adolescents engage in digital communication with their parents. However, a thorough theoretical and methodological understanding of the interplay between parent‐adolescent digital communication and relationship quality is still needed to provide a foundation for future research on how to conceptualize, measure, and examine these associations. To begin to address this gap, this systematic review was designed to synthesize empirical literature on the role of digital technology in the parent‐adolescent relationship, focusing on families residing together in the U.S. Through a systematic evaluation of research quality and a synthesis of studies, current gaps in the literature were identified, providing suggestions for future research.

### 3.1 Foundational Theories and Conceptual Frameworks

The reviewed studies drew on a range of theoretical and conceptual frameworks spanning family science, psychology, media, and communication studies, and each highlighted a different facet of the phenomenon. Family theories situated digital communication within the broader context of parent‐adolescent relational interactions, while parenting frameworks highlighted digital communication as a tool through which parenting is enacted. Additionally, media and communication theories emphasized how the properties of digital devices shape the communication they support. This disciplinary diversity is a strength, allowing scholars in the field to examine how digital communication relates to relationship quality across different contexts and from different perspectives. At the same time, researchers rarely draw on frameworks from other disciplines, missing the added insights that an interdisciplinary approach could offer. Such integration can simultaneously account for the range of communication modalities and the rapidly shifting societal context, such as the saturation of digital communication in family life and the continual evolution of parent‐adolescent relationships in the digital age.

Scholars have begun this integrative work. For example, Dworkin et al. (2019) combined family and media theories into a more nuanced framework for understanding communication technology in parent‐adolescent relationships, considering the developmental transition of adolescence and identifying factors (e.g., complicated power dynamics, connecting and distancing) that shape both family relationships and digital communication. Similarly, Lanigan (2009) modeled the interplay among technology characteristics, individual traits, family factors, and extrafamilial influences, capturing both how information and communication technologies affect families and how families shape their use. These frameworks illustrate what interdisciplinary integration can offer: a more comprehensive account of how digital technologies are woven into family interactions across different developmental stages and relational contexts. Yet such efforts remain the exception rather than the norm in the empirical work reviewed here; studies more often draw on a single disciplinary lens.

### 3.2 Measures of Digital Communication and Relationship Quality

#### 3.2.1 Conceptualization

Our synthesis points to a need for researchers to make their conceptualization of digital communication more explicit, because the way the construct is defined shapes how its associations with relationship quality are interpreted. Across the reviewed studies, digital communication encompassed multiple modalities, including texting, calls, and social media, and was characterized along several dimensions, such as frequency, quantity, and reasons for use (see Table 4). These varied conceptualizations were mirrored in the measures: approaches ranged from objective indicators, including cell phone histories, to a self‐reported survey asking participants to recall their recent communication. This heterogeneity is consequential. The mixed findings we observed, where digital communication could either foster closeness or generate conflict depending largely on its purpose, are difficult to reconcile when studies define and measure the construct differently. A clearer and more consistent conceptualization across studies would support more comparable measurement and help reduce these discrepancies. It would also provide firmer ground for practitioners, enabling more concrete guidance for parents and adolescents on using digital communication to strengthen their relationships.

#### 3.2.2 Measurement

This review also revealed variation in how studies measure digital communication. Measuring a single dimension, such as frequency, is insufficient to fully capture how adolescents and their parents integrate digital communication into their relationship or how it shapes the quality of that relationship. This aligns with the tenets of uses and gratification theory (Ruggerio 2000), which posits that adolescents select the most suitable modalities of digital communication to fulfill their needs effectively. From this perspective, the intent and content of an exchange, not merely its frequency, shape how adolescents experience and interpret their communication with parents and, in turn, their sense of connectedness and support. Whether an interaction is driven by emotional support, coordination, conflict, or a routine check‐in may influence relationship quality in different ways. Consistent with this, a 15‐day diary study found that adolescents' digital communication with parents is shaped by various topics, including school, sports, monitoring activities, and family issues (Janssen et al. 2025). To fully understand parent‐adolescent digital communication, measures of digital communication should be more specific, and the reasons adolescents choose to use digital communication should be examined.

Across studies, relationship quality was measured using frameworks that draw on cognitive, emotional, or both dimensions of the relationship. The cognitive dimension typically reflects perceptions, beliefs, and appraisals of the relationship, whereas the emotional dimension encapsulates affective experiences and responses within relational contexts. Interestingly, while all reviewed studies grounded their work in one or both of these dimensions, each employed a distinct measure of relationship quality, the only exceptions being studies drawing on the same dataset (Jensen et al. 2021a, 2021b). This variation in measures may be attributed to differences in theoretical perspectives that influence underlying conceptual and methodological approaches. Regardless, this measurement variability makes findings difficult to compare and synthesize across studies, compounding the inconsistency already introduced by varied conceptualizations of digital communication. Refining how relationship quality is conceptualized is, therefore, as crucial as refining how digital communication is measured.

### 3.3 Interpreting the Role of Digital Communication in Parent‐Adolescent Relationship Quality

Across studies, it is clear that the association between digital communication and parent‐adolescent relationship quality is fundamentally shaped by the purposes communication serves, the platforms on which it occurs, and the relational dynamics it reflects. The inconsistent associations reported in this literature may stem largely from frequency‐based measures, which aggregate exchanges that serve different purposes and can pull relationship quality in opposite directions. Counting only how often adolescents and parents communicate conflates interactions that strengthen bonds (e.g., sharing positive updates) with those that signal or amplify relational problems. Consistent with this, more frequent communication specifically for planning and sharing materials (Aloia and Warren 2019), handling conflicts (Warren and Aloia 2018), and managing parental controls (Jensen et al. 2021a) was negatively associated with relationship quality, suggesting that instrumental or tension‐laden purposes may exacerbate rather than alleviate relational strain. A frequency‐only approach may therefore yield inconsistent results because it merges communication with positive relational functions and communication reflecting underlying difficulty. Future research should move beyond simple frequency to examine the communicative intent and relational context underlying digital exchanges. Disaggregating the data by purpose would also help frame complex research questions, such as whether digital communication genuinely shapes relationship quality or merely reflects pre‐existing relational dynamics.

Differences across communication modalities provide additional insight into how digital communication influences the quality of parent‐adolescent relationships. Text messaging has shown fairly consistent positive associations (Manago et al. 2020; Padilla‐Walker et al. 2012; Vaterlaus et al. 2019), whereas social media has exhibited more complex patterns, with negative associations being particularly evident in father‐adolescent relationships (Vaterlaus et al. 2019). These differences likely reflect the distinct affordances each platform provides. For example, text messaging provides a private, dyadic channel in which adolescents can maintain a connection with their parents while controlling the timing, intensity, and visibility of the interaction, whereas social media interactions occur in public spaces where peer audiences may witness parent‐adolescent exchanges. The modality‐specific patterns thus suggest adolescents are not passive recipients of communication but active agents (Dworkin 2018) who navigate platforms strategically to balance closeness with autonomy. Understanding how the platforms shape relationship quality, therefore, requires attention to the structural features of each modality and to how they enable or constrain parent‐adolescent connection.

Further, fathers' relationships appear sensitive to how digital communication unfolds, and this pattern may echo across studies for different reasons. Hessel et al. (2017) found that fathers' online solicitation through a child's friends was linked to worse adjustment, which they attributed to adolescents experiencing it as a privacy violation. Keyzers and Dworkin (2023) found that fathers' approval of online behavior was most strongly tied to monitoring and adolescent online behavior within father‐son dyads, which they linked instead to fathers' greater familiarity with sons' daily lives. Together, these findings point to father‐son dyads as a context in which fathers' digital involvement may carry distinct relational weight.

A further gap concerns device access. Current research on parent‐adolescent digital communication has largely overlooked whether, and for how long, adolescents have owned their own digital devices that enable and facilitate digital communication. Only one study (Weisskirch 2011) considered adolescents' cell phone ownership (i.e., adolescents had to have owned a cell phone for at least 6 months). Duration of ownership may matter because it can shape both how often adolescents communicate digitally and the purposes behind it. This is supported by studies examining adolescents' digital communication, which have shown that smartphone ownership provides a critical context for understanding the nature and impact of digital interactions with parents (e.g., Moreno et al. 2019; Vaterlaus et al. 2021). This temporal dimension is especially relevant given that parent‐adolescent communication patterns develop through accumulated interaction over time (Branje 2018). Accordingly, digital communication habits across modalities may likewise take time to form and stabilize. Future research should therefore consider how long adolescents have owned their devices, allowing a more nuanced view of how digital communication shapes parent‐adolescent dynamics as those patterns mature.

Gender within parent‐adolescent dyads also received little attention in the reviewed studies, despite evidence that it shapes digital communication in meaningful ways; adolescents tend to call and text mothers more frequently than fathers (Fletcher et al. 2018; Jensen et al. 2023). This suggests that associations between digital communication and parent‐adolescent relationship quality might operate differently across dyad types. Yet fathers remain markedly underrepresented in research relative to mothers, limiting what can be said about these differences. Future research should sample a comparable number of mother‐son, mother‐daughter, father‐son, and father‐daughter dyads, enabling direct comparison of how digital communication functions across dyads. Such work would clarify whether effective digital communication varies by dyad and, in turn, support more tailored guidance for researchers and parents, grounded in the specific nature of their relationship with their adolescent.

### 3.4 Limitations and Future Directions

While this review was designed to identify gaps in the field, it has limitations. Studies included in this review were published from 2011 to 2024. However, due to rapid changes in digital communication, the body of literature on this topic is evolving quickly, and studies conducted earlier in this window may reflect a different media environment than those conducted more recently. New, timely, and sensitive literature continues to emerge, providing findings and implications that advance this field. For instance, more research should examine societal shifts, such as those related to COVID‐19, which have altered how families utilize digital communication (Kalenkoski and Pabilonia 2024). Findings may differ regarding how, and to what extent, adolescents use digital communication with their parents before and after the COVID‐19 pandemic. Additionally, this systematic review employed specific terms to encompass various operationalizations of digital communication. Due to the terms used in the databases searched, it is possible that some studies may not have emerged through this search process. However, the thorough screening process reduces the likelihood of this. In addition, most studies included in this review are quantitative, cross‐sectional studies. This limits our understanding of adolescents' developmental processes in both a narrative (qualitative) way that considers adolescents' and their parents' perspectives and a longitudinal perspective on parent‐adolescent digital communication and relationship quality. Relying mainly on quantitative data also risks overstating what can be concluded about why families use certain digital communication methods or what these patterns mean for their relationships. Further research should consider qualitative and longitudinal study designs to enhance insights into the use of digital communication in parent‐adolescent relationships across different developmental periods. Finally, this review was intentionally limited to U.S. adolescents and their parents living together. Given that cultural factors vary substantially across international contexts and meaningfully shape how digital communication unfolds within families, the findings may not be directly generalizable to families in other cultural or international contexts. Future research should explore parent‐adolescent digital communication and relationship quality across diverse cultural contexts (Rogers et al. 2021) to broaden our understanding of how these dynamics function within varying sociocultural environments.

## 4 Conclusion

This comprehensive systematic review examined the current empirical evidence on parent‐adolescent digital communication and its association with relationship quality, focusing on U.S. adolescents living with their parents. This specific population represents a distinctive context for understanding digital communication in family relationships, where it serves a supplementary role, bridging physical and emotional distance and supporting relationship maintenance beyond face‐to‐face exchange. Across the reviewed studies, both digital communication and relationship quality were conceptualized through varied theoretical lenses and measured differently, a heterogeneity that helps explain the mixed findings in the literature. A clear pattern nonetheless emerged: the purpose and platform of digital communication matter more than frequency alone. Therefore, future research should move beyond frequency to examine the communicative intent and relational context of digital exchanges, while drawing on interdisciplinary frameworks that integrate family, media, communication, and developmental perspectives. This review further points to the value of accounting for how long adolescents have owned their devices and for gender dynamics within dyads, both of which appear to shape digital communication practices and their relational outcomes. Ultimately, advancing this field requires frameworks and measures that capture the complexity of digital communication in family life, moving toward ecologically valid, contextually grounded insights that can inform both theory and practice.

## Author Contributions

**Eunyoung Park:** conceptualization, writing – original draft, writing – review and editing, investigation, methodology, visualization. **Jodi Dworkin:** supervision, writing – review and editing, methodology, investigation.

## Funding

The authors have nothing to report.

## Ethics Statement

Ethical approval was not required for this systematic review as all data were extracted from already published studies.

## Conflicts of Interest

The authors declare no conflicts of interest.

## Data Availability

Data sharing is not applicable to this article, as no data sets were generated or analyzed during the current study. The full search strategies and PRISMA flow diagram are provided in the manuscript.
